# Immunomodulatory Properties of *Leishmania* Extracellular Vesicles During Host-Parasite Interaction: Differential Activation of TLRs and NF-κB Translocation by Dermotropic and Viscerotropic Species

**DOI:** 10.3389/fcimb.2020.00380

**Published:** 2020-07-29

**Authors:** Paula Monalisa Nogueira, Armando de Menezes-Neto, Valéria M. Borges, Albert Descoteaux, Ana Claudia Torrecilhas, Patrícia Xander, Or-Yam Revach, Neta Regev-Rudzki, Rodrigo Pedro Soares

**Affiliations:** ^1^Instituto René Rachou, Fundação Oswaldo Cruz - FIOCRUZ, Belo Horizonte, Brazil; ^2^Instituto Gonçalo Moniz, Fundação Oswaldo Cruz - FIOCRUZ, Salvador, Brazil; ^3^INRS - Centre Armand-Frappier Santé Biotechnologie, Université du Québec, Laval, QC, Canada; ^4^Departamento de Ciências Farmacêuticas, UNIFESP, Diadema, Brazil; ^5^Department of Biomolecular Sciences, Weizmann Institute of Science, Rehovot, Israel

**Keywords:** extracellular vesicles, *Leishmania*, host-parasite interaction, innate immunity, lipophosphoglycan (LPG)

## Abstract

*Leishmania* infection causes considerable human morbidity and may develop into a deadly visceral form in endemic regions. The parasite infects macrophages where they can replicate intracellularly. Furthermore, they modulate host immune responses by using virulence factors (lipophosphoglycan, glycoprotein-63, and others) that promote survival inside the cells. Extracellular vesicles (EVs) released by parasites are important for cell-cell communication in the proinflammatory milieu modulating the establishment of infection. However, information on the ability of EVs from different *Leishmania* species to modulate inflammatory responses is scarce, especially from those species causing different clinical manifestations (visceral vs. cutaneous). The purpose of this study was to compare macrophage activation using EVs from three *Leishmania* species from New World including *L. infantum, L. braziliensis*, and *L. amazonensis*. EVs were released from promastigote forms, purified by ultracentrifugation and quantitated by Nanoparticle Tracking Analysis (NTA) prior to murine macrophage exposure. NTA analysis did not show any differences in the EV sizes among the strains. EVs from *L. braziliensis* and *L. infantum* failed to induce a pro-inflammatory response. EVs from both *L. infantum* WT and LPG-deficient mutant (LPG-KO) did not show any differences in their interaction with macrophages, suggesting that LPG solely was not determinant for activation. On the other hand, EVs from *L. amazonensis* were immunomodulatory inducing NO, TNF-α, IL-6, and IL-10 via TLR4 and TLR2. To determine whether such activation was related to NF-κB p65 translocation, THP-1 macrophage cells were exposed to EVs. In the same way, only EVs from *L. amazonensis* exhibited a highly percentage of cells positive for NF-κB. Our results suggest an important role of EVs in determining the pattern of immune response depending on the parasite species. For *L. infantum*, LPG was not determinant for the activation.

## Introduction

Leishmaniasis is a spectrum of human diseases caused by the protozoan parasite *Leishmania* and transmitted by sandflies. Annually, an estimated 1.3 million new cases and around 30,000 deaths are associated with these diseases (WHO, 2016). There is currently no effective and well-tolerated vaccine and treatments are difficult and toxic. Clinical manifestations associated with Leishmaniasis range from self-healing ulcers to lethal visceral form (VL), and depend on parasite species and the effective host immune response (Desjeux, 2004). In Latin America, especially in Brazil, VL is caused by *Leishmania infantum*, the potentially fatal infection is due to the systemic spread of the parasite and whose symptoms are characterized by progressive chronic fever, hepatosplenomegaly and pancytopenia. *Leishmania braziliensis* is the major cause of the most common form of cutaneous leishmaniasis (CL), and less frequent the mucosal leishmaniasis (MCL). Additionally, *Leishmania amazonensis* may cause different cutaneous forms ranging from regular CL to multiple non-ulcerative lesions, known as diffuse cutaneous leismaniasis (DCL) often resistant to antimonial chemotherapy (Herwaldt, 1999; Silveira et al., 2009). More importantly, most mechanisms underlying the events responsible for those different clinical forms are unknown but may be related to specific virulence factors from the parasites.

*Leishmania* has developed strategies to evade or subvert macrophage microbicidal effector mechanisms. Inhibition of macrophage activities may be induced by a range of *Leishmania*-derived virulence factors. In the New World, Lipophosphoglycan (LPG) is a highly abundant surface molecule and it has been shown to interfere with macrophage signaling through generation of nitric oxide (NO), cytokines, and MAPKs (Ibraim et al., 2013; Nogueira et al., 2016). Moreover, LPG-defective *L. infantum* promastigotes (Δ*lpg1*) displayed reduce ability to replicate inside murine macrophages and induced a robust *iNOS* expression compared to parental WT, supporting the status of LPG as a virulence factor (Lázaro-Souza et al., 2018). Interspecies polymorphisms in the LPG structure are important during host immune responses, and may explain, at least in part, the differences in the immunopathology. In murine macrophages, *L. braziliensis*/*L. amazonensis* LPGs were more pro-inflammatory than that *L. infantum* via TLR4 and TLR2 (Ibraim et al., 2013; Nogueira et al., 2016; Vieira et al., 2019). Together with GP63, LPG is also a virulence factor found in the EVs from *Leishmania* (Barbosa et al., 2018). *Leishmania* parasites and/or EVs can modulate macrophage transcription factors in an LPG and GP63-dependent manner (Silverman et al., 2010b; Hassani et al., 2014; Atayde et al., 2015). However, a wide range of molecules found in EVs structures are crucial for orchestrating the interaction between a given pathogen with its host (Szempruch et al., 2016).

Most studies using *Leishmania* EVs involved Old World *Leishmania* such as VL species *Leishmania donovani*. An interesting feature of *Leishmania* EVs was the presence of the zinc metalloprotease GP63. Functionally, *L. donovani* EVs were found to be anti-inflammatory modulating cytokines expression at the site of infection (Silverman et al., 2010a,b). This modulation could be due to IL-17 production and IL-1β decrease impairing NLRP3 inflammasomes (Atayde et al., 2015).

Recently, EVs isolate from *L. amazonensis* were shown to modulate immune responses in B-1 cells by inducing the production of IL-6 and TNF-α and by inhibiting IL-10 (Barbosa et al., 2018). However, how those mechanisms contribute to the severity of the disease in New World species of *Leishmania* is still unknown. Recognizing the importance of glycoconjugates and EVs in pathogenesis of Leishmaniasis, the purpose of this study was to compare their effect during macrophage activation in the innate immune compartment.

## Experimental Procedures

### Ethics Statement

All animals were handled in strict accordance with animal practice as defined by the Internal Ethics Committee in Animal Experimentation (CEUA) of Fundação Oswaldo Cruz (FIOCRUZ), Belo Horizonte, Minas Gerais (MG), Brazil (protocol P-17/14-2). This protocol followed the guidelines of CONCEA/MCT. Knock-out mice handling protocol was approved by the National Commission on Biosafety (CTNBio) (protocol no. #01200.006193/2001-16).

### Parasites

Promastigotes *L. infantum* (MCAN/BR/89/BA262), *L. braziliensis* (MHOM/BR/01/BA788), and *L. amazonensis* (MHOM/BR/87/BA125) species were cultured in M199 medium supplemented with 10% fetal bovine serum (FBS) 26°C. The *L. infantum* BA262 LPG-deficient mutant (Δ*lpg1*) (LPG-KO) was cultured in the same medium, supplemented with Hygromycin (50 μg/mL) and G418 (70 μg/mL) (Lázaro-Souza et al., 2018). After the 6th day, stationary phase parasites were washed in PBS (1,000 *g*, 15 min) and Hanks' balanced salt solution (HBSS) and incubated for 2 h in M199 without FBS under agitation (cell density of 1 × 10^8^ cells/mL) (37°C, 5% CO_2_) for EVs release.

The viability of parasites was assessed by Trypan blue 0.4% exclusion (Gibco). Cultures were centrifuged (1,000 g for 15 min) and parasites from the pellet were fixed in 2.5% glutaraldehyde in 0.1 M phosphate buffer, pH 7.2, for scanning electron microscopy (SEM). Fixed parasites were post fixed with 1% osmium tetroxide, treated with tannic acid (0.1%), and dehydrated with ethanol. Samples were observed in a Field Emission FEI Quanta 250 FEG scanning electron microscope (FEI, OR, USA), as described (Nogueira et al., 2015).

For functional assays, supernatants were filtered (0.22 μm sterile vacuum filter) to remove residual cells and debris, 3 mL of PBS was added and the solution ultracentrifuged (100,000 *g* 4°C, 2 h) for EVs isolation. EVs were quantitated by NTA and resuspended in RPMI medium without FBS to a final concentration of 1 × 10^8^ particles/mL. Protein concentration was determined using the Micro BCA proteins assay kit (Thermo Scientific Waltham, MA).

### Nanoparticle Tracking Analysis (NTA)

All the batches collected were pooled. Size, distribution and concentration of isolated particles were measured in a Nanosight NS300 instrument (Malvern Instruments Ltd, Malvern, UK) equipped with a 405-nm laser and coupled to a CCD camera (the laser emitting a 60-mW beam at 405-nm wavelength), and data were analyzed using the NTA software (version 2.3 build 0017). The detection threshold was set to 10. Blur, Min track Length, and Min Expected Particle Size were set to auto. To perform the measurements, each sample diluted (1:100) in PBS was analyzed in triplicate; and loaded into the instrument for 30 s at 20 frames per second with the camera level set to 14 and manual monitoring of temperature (20°C).

### Purification of Murine Peritoneal Macrophages

Thioglycollate-elicited peritoneal macrophages were isolated from C57BL/6 and C57BL/6 (TLR2–/– and TLR4–/– knockouts) by peritoneal washing with ice cold serum-free RPMI and enriched by plastic adherence for 1 h at 37°C/5% CO_2_. Cells (3 × 10^5^ cells/well) were washed with fresh RPMI then cultured in RPMI, 2 mM glutamine, 50 U/ml of penicillin and 50 μg/mL streptomycin supplemented with 10% FBS in 96-well culture plates (37°C/5% CO_2_). Cells were primed with Interferon-γ (IFN-γ) (0.5 ng/mL) for 18 h prior to incubation with EVs (1 × 10^8^ particles/mL) from *L. infantum* (WT and LPG-KO), *L. braziliensis*, and *L. amazonensis*. A positive control included lipopolysaccharide from *Escherichia coli* (LPS–TLR4+) (100 ng/mL) and extract of *Staphylococcus aureus* (Sa–TLR2+) (100 ng/mL). Negative control included medium with IFN-γ.

### Cytokines and Nitrite Measurements

The cytokines TNF-α, IL-6, and IL-10 were determined using BD CBA Mouse Cytokine Assay kits according to the manufacturer's specification (BD Biosciences, CA, USA). Flow cytometry measurements were performed on a FACSCalibur flow cytometry (BD Bioscience, CA, USA) collected using the Cell-QuestTM software package provide by the manufacturer (1200 events). FlowJO software 7.6.4 (Tree Star, Inc, Ashland, OR) was used for data analysis. Results are representative of two experiments in triplicate. Nitrite concentration (NO) was determinate by Griess Reaction as described (Nogueira et al., 2015).

### NF-κB Activation Assay

THP-1 (ATCC® TIB-202™) monocyte cells were differentiated into macrophages by phorbol 12-myristate 13-acetate (PMA) for 3 days (Cells were tested for *Mycoplasma* contamination by using the polymerase chain reaction (PCR) methodology (Uphoff et al., 2002). Cells were cultured on gelatin coated glass bottom plates (Cellvis) (1 × 10^6^ cells/well) and incubated for 1 h, following incubation with EVs (1 × 10^8^ particles/mL) from different *Leishmania* species for another 1 and 6 h. Cells were fixed with 4% Paraformaldehyde (PFA), blocked with 5% bovine serum albumin (BSA) and stained with p65 antibody (IgG monoclonal–Santa Cruz) and Hoechst (H33342 Molecular Probes Ltd.−1 ng/mL). Images were acquired using the DeltaVision microtiter system (Applied Precision, Inc.), using a 40 × /1.3 oil objective (Olympus). Image analysis was performed using ImageJ software (rsbweb.nih.gov/ij). In each sample more than 100–200 cells were quantified for cytoplasmic or nuclear localization of p65 (Sisquella et al., 2017; Ofir-Birin et al., 2018).

### Statistical Analyses

For nitrite and cytokine measurements the Shapiro Wilk test was conducted to test the null hypothesis that data were sampled from a Gaussian distribution. In this case, student's *t*-test was performed. For the non-parametric distribution, it was performed the Mann-Whitney test. Data were analyzed using GraphPad Prism 5.0 software (Graph Prism Inc., CA, USA). *P* < 0.05 was considered significant.

## Results

### Characterization of EVs Isolated From Different *Leishmania* Species

**Figure 1:**
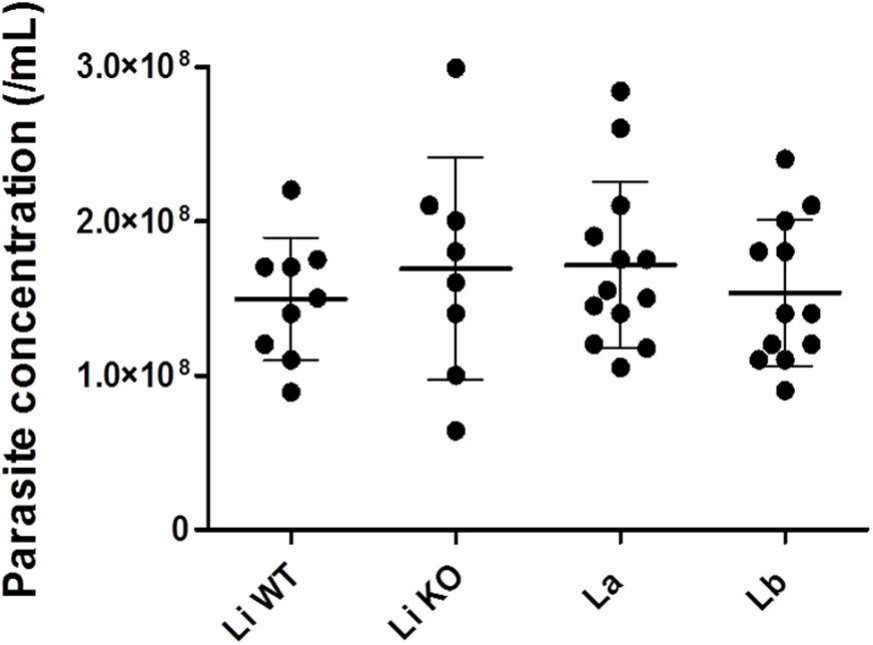
*Leishmania* promastigotes concentrations for EVs release. Statistical analysis was performed based on Mann-Whitney test (*P* > 0.05).

**Figure 2:**
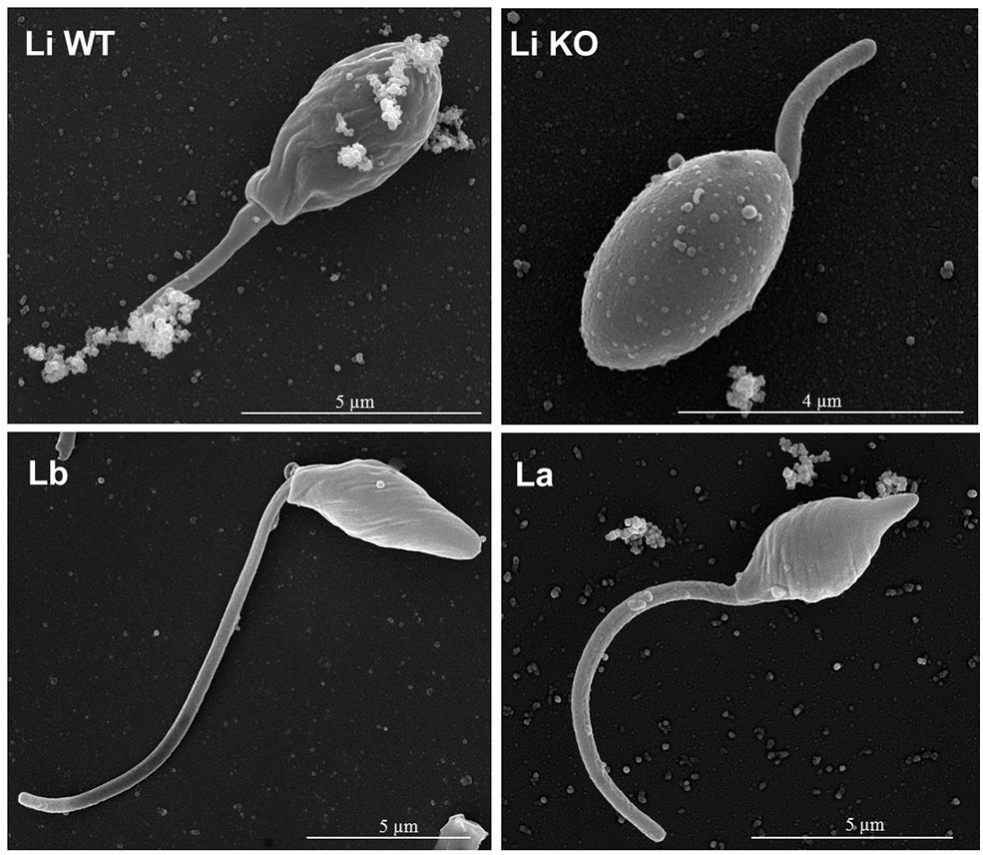
Representative SEM images of *Leishmania* structure and release EV from entire parasite surface. Each panel shows promastigotes pre-incubated in RPMI and attached to glass coverslips containing poly-L-lysine obtained from the *L. infantum* (WT and KO), *L. braziliensis*, and *L. amazonensis* fixed and processed for SEM. The bar sizes are indicated in each image. Li WT = EVs from *L. infantum*; Li KO = EVs from *L. infantum* LPG-KO; Lb = EVs from *L. braziliensis*; La = EVs from *L. amazonensis*.

Parasites were grown in a simple serum-free media to ensure that EVs were from *Leishmania* origin. EVs were released by 1–2 × 10^8^ parasites/mL (Figure 1) and viability was above 90% (data not shown) after 2 h in incubation. Those parasites were fixed and subjected to SEM. All promastigote forms exhibited the expected shapes and microscopic images off all strains/species revealed shedding of EVs from cellular body and flagellum of the parasite (Figure 2).

**Figure 3:**
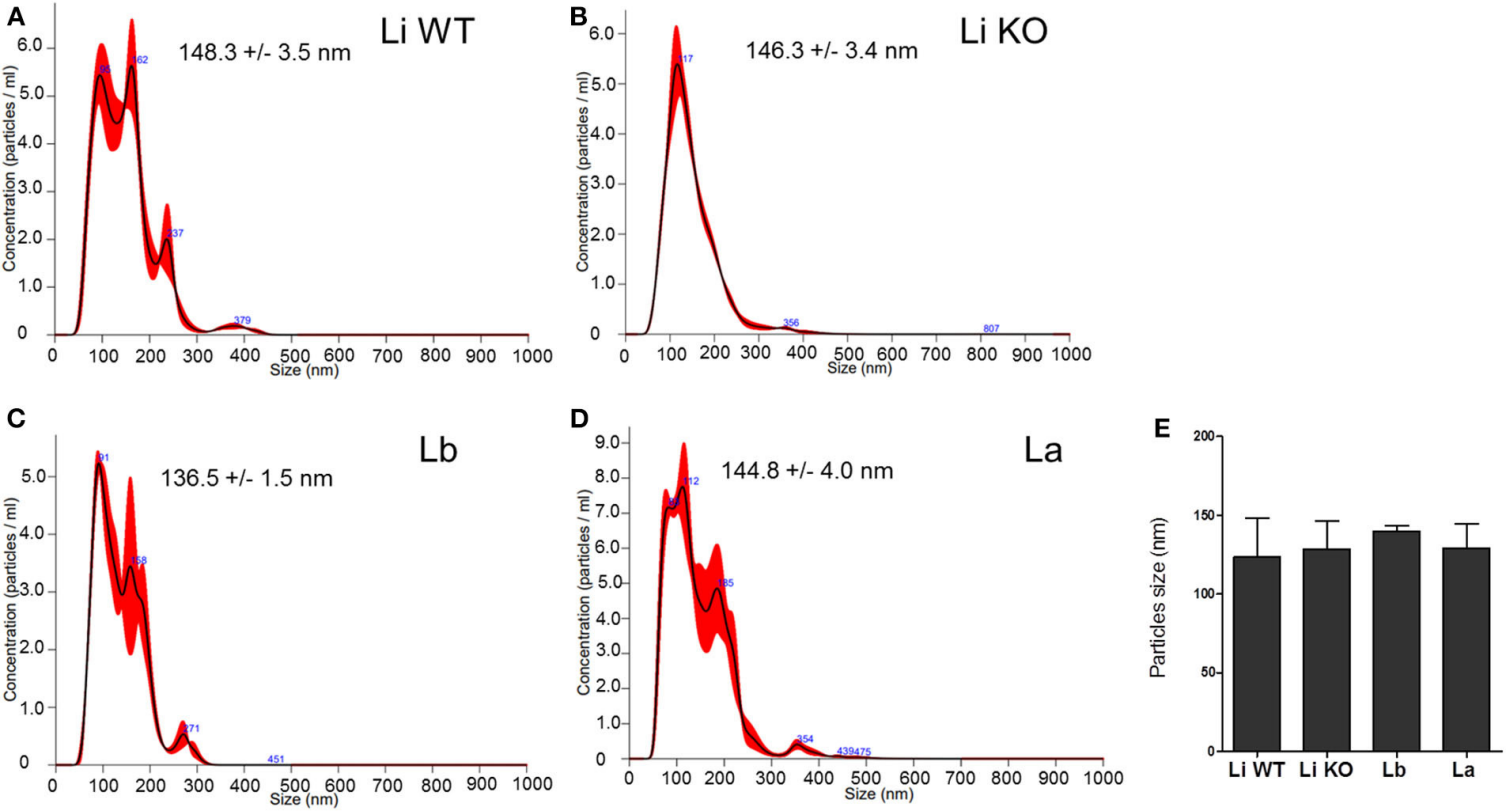
EVs size (nm) determined by NTA analysis. Sample size distributions were calibrated in a liquid suspension by the analysis of Brownian motion via light scattering. Nanosight provides single particle size and concentration measurements. Readings were taken in triplicates. Averaged Finite Track Length Adjustment (FTLA) concentration/size for experiment. The red line is error bars indicate ±1 standard error of the mean. **(A)** Li WT = EVs from *L. infantum*; **(B)** Li KO = EVs from *L. infantum* LPG-KO; **(C)** Lb = EVs from *L. braziliensis*, and **(D)** La = EVs from *L. amazonensis*. **(E)** This figure represents a typical analysis of average EV sizes from at least two independent experiments. Statistical analysis was performed based on Mann-Whitney test (*P* > 0.05).

NTA analysis is a very important way to quantitate EVs from several cell types. The EVs exhibited mean diameter sizes of 123.5 ± 3.5 and 128.5 ± 3.4 nm for *L. infantum* (WT and LPG-KO), 140.0 ± 1.5 nm for *L. braziliensis*, and 129.0 ± 4.0 nm for *L. amazonensis* (Figures 3A–D). Statistical analysis did not show differences any in the average sizes according to species and strains (*P* > 0.05, Figure 3E). As expected, no particles were found in the medium alone (negative control, data not shown).

### *Leishmania* EVs Differentially Activate TLRs

**Figure 4:**
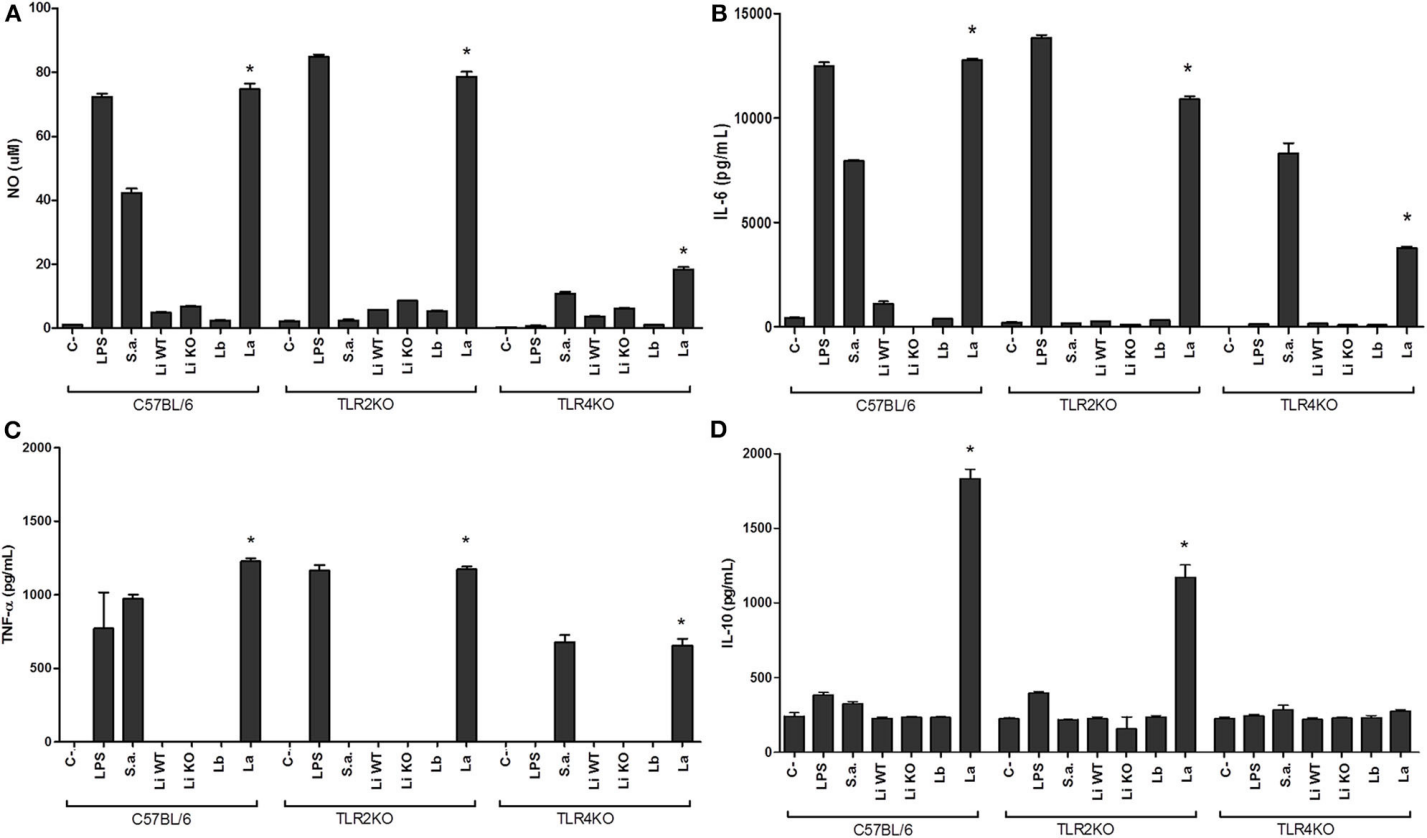
NO and cytokines production by IFN-γ primed murine macrophages stimulated with EVs from different *Leishmania* species. Supernatants were used for NO **(A)** and cytokines IL-6 **(B)**, TNF-α **(C)**, and IL-10 **(D)** measurements were collected 48 h latter. Nitrite concentration was measured by Griess reaction. TNF-α, IL-6, and IL-10 levels were determined by CBA analysis. Li WT = EVs from *L. infantum*; Li KO = EVs from *L. infantum* LPG-KO; Lb = EVs from *L. braziliensis*; La = EVs from *L. amazonensis*. Statistical analysis was performed based on student's *t*-test (*P* > 0.05). *Asterisks indicate statistical differences.

Toll-like receptors are very important molecules involved in the recognition of different pathogen-associated molecular patterns (PAMPs). It is already reported that EVs from *T. cruzi* carry important PAMPs involved in the activation of TLR2 (Nogueira et al., 2015). Since LPGs from *Leishmania* species are polymorphic and differentially modulate the immune system (Ibraim et al., 2013; Nogueira et al., 2016), the ability of their respective EVs to induce NO and TNF-α, IL-6, and IL-10 cytokines in murine macrophages was investigated. We have used knock-out mice lacking TLR2 and TLR4 receptors in order to ascertains the type of recognition by EVs. As expected, the controls represented by LPS (TLR4) and *S. aureus* (TLR2) activated TLR2KO and TLR4KO macrophages, respectively. In the wild type C57BL/6, those agonists activated both TLRs. *Leishmania infantum* and *L. braziliensis* EVs induced lower amounts of NO, TNF-α, IL-6, and IL-10 than those from *L. amazonensis* (*p* < 0.05) (Figures 4A–D). No differences were observed between EVs from *L. infantum* WT and LPG-KO (*p* > 0.05). Activation of macrophage immunomodulatory responses was markedly increased by EVs from *L. amazonensis* and this was primarily mediated by TLR4 and secondarily via TLR2 (Figures 4A–D).

**Figure 5:**
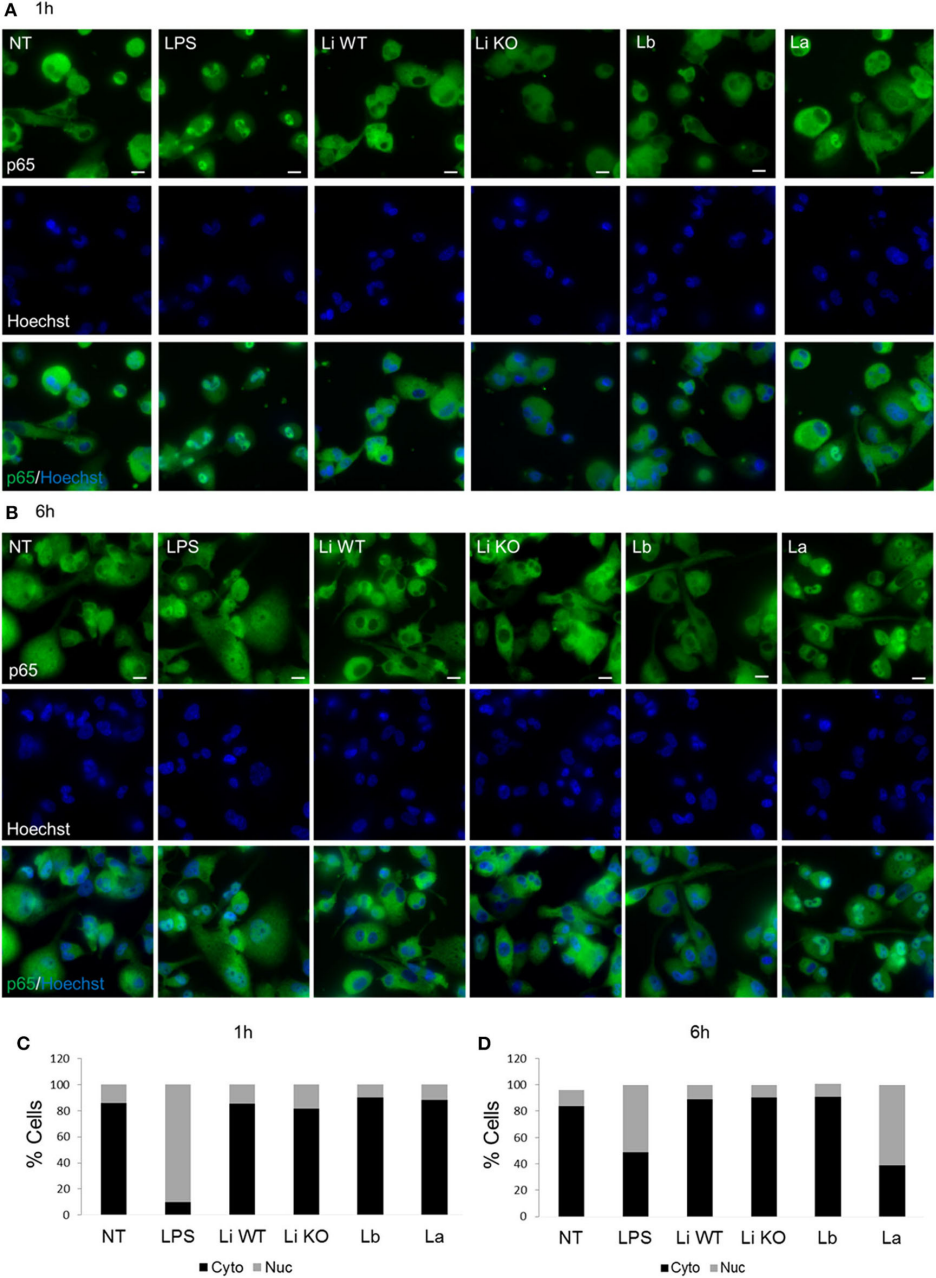
THP-1 macrophages were treated for 1 h **(A)** and 6 h **(B)** with EVs from different *Leishmania* species to evaluate the nuclear translocation of NF-κB p65. Cells were stained with p65 (green) and Hoechst (blue) and an overlay of the two images are presented. Quantification of percentage of cells with cytoplasmic or nuclear localization of p65 for1 h **(C)** and 6 h **(D)**. NF-κB p65 fluorescence was evaluated by intranucleus intensity (the sum of the background-subtracted pixel values within the masked area of the image) and the max pixel (the largest value of the background subtracted pixel) as previously reported (Sisquella et al., 2017).

To confirm whether EV-induced cytokine production was associated with NF-κB p65 nuclear translocation, THP-1 macrophages cells were exposed to EVs from all *Leishmania* species/strains for 1 and 6 h and the percentage of positive cells was evaluated. This nuclear factor is important for inducing cytokine production in the innate immune compartment. This evaluation is based on the green fluorescence detected inside the nucleus. With exception to LPS (positive control), no significant nuclear translocation of the NF-κB p65 for all *Leishmania* species was detected after 1 h (Figures 5A,C). As expected, after 6 h a decrease in nuclear NF-κB was observed for LPS (positive control). Different from *L. braziliensis* and *L. infantum* (WT and LPG KO), only *L. amazonensis* EVs were able to induce the nuclear translocation of NF-κB among *Leishmania* species after 6 h of exposure (Figures 5B,D). Those results confirmed the higher pro-inflammatory ability of *L. amazonensis* EVs in the NO and cytokine measurements (Figures 4A–D).

## Discussion

Extracellular vesicles are cell-derived particles released from a variety of cell types, including pathogens and infected cells. These structures are recognized as important mediators of cell-cell communication, immune regulation and dissemination of pathogenic material (Campos et al., 2015). EVs possess a cargo of several biomolecules including proteins, lipids, glycoconjugates, metabolites, DNA, mRNA, microRNA, and other non-coding RNA species (Mathieu et al., 2019). Secretion of EVs has been reported in several pathogens such as *Leishmania, Trypanosoma, Toxoplasma, Plasmodium, Trichomonas*, and *Mycobacterium*. Thus, the importance of those structures during the interaction with the immune system has been extensively explored (Regev-Rudzki et al., 2013; Szempruch et al., 2016).

Several research groups have focused on the study of extracellular vesicles in *Leishmania* especially in their role during cell-cell communication and innate immune responses (Atayde et al., 2016; Dong et al., 2019). Recently, EVs were shown to be important for LRV1 release in *Leishmania guyanensis* reinforce their role as potential in increasing severity in cutaneous leishmaniasis (Atayde et al., 2019). The recovered *Leishmania* EVs from New World species did not differ in size, were below 1,000 nm and viability was higher than 90%, indicating they were free of apoptotic bodies (Silverman et al., 2010a; Atayde et al., 2016; Barbosa et al., 2018). Also, those data were confirmed by SEM showing EVs being released from entire parasite surface in concentrations detected by NTA, consistent with the size and morphology of exosomes released by others parasites.

Healing of Leishmaniasis requires an effective immune response capable of eliminating the parasites with minimal damage of tissues. As part of sand fly inoculum, EVs influence the early events in the establishment of the parasite infection, play a role in pathogenesis by modulating cytokines/chemokines at the site of infection (Silverman and Reiner, 2011; Atayde et al., 2015). Cytokines such as IFN-γ and TNF-α are important activators of macrophage parasite killing whereas IL-10 participates in the control of immunopathology (Gazzinelli and Denkers, 2006). Functionally, early studies have shown that viscerotropic *L. donovani* EVs are anti-inflammatory (Silverman et al., 2010b). Here, with another viscerotropic species (*L. infantum*), no activation was detected upon macrophage stimulation by its EVs. It has been reported that those immunomodulatory and signaling-inducing activities are due to the presence of parasitic virulence factors like GP63 (Silverman et al., 2010b; Hassani et al., 2014; Marshall et al., 2018). Exosomes from WT *L. major* differentially modulated the induction of transcription factors compared to an isogenic *L. major* gp63 KO (Hassani et al., 2014). Interestingly, in our experiment, the lack of LPG in EVs from *L. infantum* did not affect macrophage activation.

Recently, EVs from *L. infantum* induced IL-10 and reduced the production of IL-18, suggesting their role in creating a permissive environment for parasite establishment (Castelli et al., 2019). Also, injection of *L. infantum* EVs increased the pro-inflammatory burden resulting in higher parasite loads (Pérez-Cabezas et al., 2018). Under our experimental conditions, EVs from *L. infantum* were also able to induce IL-10 confirming previous findings (Castelli et al., 2019). However, the presence/absence of LPG from *L. infantum* EVs did not affect their ability to induce NO and cytokine production, suggesting that other molecules could explain at least in part this lack of activation. Several EVs molecules in *L. infantum* EVs have been described (Santarém et al., 2013). However, glycoconjugates have been neglected in such approaches. The presence of other potent anti-inflammatory molecules, like glycoinositolphospholipids (GIPLs) (Assis et al., 2012) could have contributed for this lack of activation. Consistent with these observations, extracellular products (EVs or EV-depleted) were shown to act together for immunomodulating host-parasite interaction in *L. infantum* both *in vitro* and *in vivo* (Pérez-Cabezas et al., 2018). However, this activation by LPG may be determinant for *L. amazonensis*. This glycoconjugate paradoxically was very pro-inflammatory/immunosuppressive via TLR4 (Nogueira et al., 2016). Interestingly, *L. braziliensis* LPG has been shown to be very pro-inflammatory (Ibraim et al., 2013; Vieira et al., 2019) via TLR2/TLR4. This feature was not observed in its EVs, which exhibited a similar pattern than those from *L. infantum*. Overall, it is very clear that interspecies polymorphisms in EVs cargo results in differential stimulation of macrophages.

Here, a remarkable activation of macrophage immunomodulatory response by *L. amazonensis* EVs was noticed compared to the other species/strains. This species is very pro-inflammatory causing ulcerated and non-ulcerated lesions. Also, it is able to disseminate causing lesions in different parts of the body associated with a lack of cellular immune response (anergy) and therapeutic failure (Silveira et al., 2009). The first studies with *L. amazonensis* showed that EVs from macrophage infected with this species were important for induction of IL-12, IL-1β, and TNF-α (Cronemberger-Andrade et al., 2014). Further, *L. amazonensis* EVs could be involved not only in the severe immunopathology of this species but also could favor dissemination of the parasites throughout the host (Barbosa et al., 2018). In this paper, co-injection with *L. amazonensis* EVs and promastigotes increased the pro-inflammatory milieu and promote a higher parasite load in the footpad. More recently, we have demonstrated that co-injection of EVs derived from B1 cells infected with *L. amazonensis* and parasites modulated iNOS and cytokine production in mouse footpads (Toledo et al., 2020). Although an increase in the parasite load and pro-inflammatory infiltrate was detected, no protective effect on lesion size was observed. Consistent with these observations, pre-treatment with *T. cruzi* EVs increased parasite invasion and spreading to larger areas in heart leading to higher mortality in mice (Trocoli Torrecilhas et al., 2009). A distinguishing feature of EVs from this species compared to *L. infantum* and *L. braziliensis*, was their ability to highly activate TLR4/TLR2 and to induce the nuclear translocation of NF-κB p65 by THP-1 monocytes. Human monocytes (THP-1 and RAW-ELAN) cells have been successfully used upon stimulation with EVs from *Plasmodium falciparum*. Alike *L. amazonensis*, EVs from the malaria parasite were also very pro-inflammatory inducing several transcriptional factors (NF-kB and IRF3) and cytokines/chemochines via TLRs (Sampaio et al., 2017; Sisquella et al., 2017; Ofir-Birin et al., 2018). Since EVs are involved in cell-cell communication, this higher ability to induce inflammation could be more exacerbated in dermotropic species. Consistent with this idea, not only EVs from *L. infantum* from our work, but also from *L. donovani*, both viscerotropic species, exhibited a more immunosuppressive profile.

In conclusion, this study shows that EVs from different *Leishmania* species were differentially recognized by murine macrophages. In contrast to GP63, LPG did not functionally affect macrophage stimulation. However, this effect was observed for an immunosuppressive viscerotropic species. Since EVs can contribute in the immunopathology, further studies are necessary for understanding the mechanism of infection, especially during its dissemination by *L. amazonensis*. EVs are remarkable structures to understand parasite-host interactions and may serve as potential novel entities for vaccine therapy and immunodiagnostics of Leishmaniasis.

## Data Availability Statement

The raw data supporting the conclusions of this article will be made available by the authors, without undue reservation.

## Ethics Statement

All animals were handled in strict accordance with animal practice as defined by the Internal Ethics Committee in Animal Experimentation (CEUA) of Fundação Oswaldo Cruz (FIOCRUZ), Belo Horizonte, Minas Gerais (MG), Brazil (protocol P-17/14-2). This protocol followed the guidelines of CONCEA/MCT. Knock-out mice handling protocol was approved by the National Commission on Biosafety (CTNBio) (protocol no. #01200.006193/2001-16).

## Author Contributions

RS, AT, PN, and AM-N: designed and performed experiments. AD and VB: development of *L. infantum* mutants. NR-R and O-YR: NF-κB translocation experiments. PX, AT, and PN: performed macrophage and CBA experiments. All authors have contributed for data analysis and writing of the manuscript.

## Conflict of Interest

The authors declare that the research was conducted in the absence of any commercial or financial relationships that could be construed as a potential conflict of interest.
